# Evaluation of four gamma-based methods for calculating confidence intervals for age-adjusted mortality rates when data are sparse

**DOI:** 10.1186/s12963-022-00288-1

**Published:** 2022-05-07

**Authors:** Makram Talih, Robert N. Anderson, Jennifer D. Parker

**Affiliations:** 1 Division of Research and Methodology, National Center for Health Statistics, Centers for Disease Control and Prevention, 3311 Toledo Road, Hyattsville, MD 20782 USA; 2 Division of Vital Statistics, National Center for Health Statistics, Centers for Disease Control and Prevention, Hyattsville, MD USA; 3 University of Porto Institute of Public Health, Porto, Portugal

**Keywords:** Direct standardization, Confidence interval width, Coverage probability, Statistical reliability

## Abstract

**Background:**

Equal-tailed confidence intervals that maintain nominal coverage (0.95 or greater probability that a 95% confidence interval covers the true value) are useful in interval-based statistical reliability standards, because they remain conservative. For age-adjusted death rates, while the Fay–Feuer gamma method remains the gold standard, modifications have been proposed to streamline implementation and/or obtain more efficient intervals (shorter intervals that retain nominal coverage).

**Methods:**

This paper evaluates three such modifications for use in interval-based statistical reliability standards, the Anderson–Rosenberg, Tiwari, and Fay–Kim intervals, when data are sparse and sample size-based standards alone are overly coarse. Initial simulations were anchored around small populations (*P* = 2400 or 1200), the median crude all-cause US mortality rate in 2010–2019 (833.8 per 100,000), and the corresponding age-specific probabilities of death. To allow for greater variation in the age-adjustment weights and age-specific probabilities, a second set of simulations draws those at random, while holding the mean number of deaths at 20 or 10. Finally, county-level mortality data by race/ethnicity from four causes are selected to capture even greater variation: all causes, external causes, congenital malformations, and Alzheimer disease.

**Results:**

The three modifications had comparable performance when the number of deaths was large relative to the denominator and the age distribution was as in the standard population. However, for sparse county-level data by race/ethnicity for rarer causes of death, and for which the age distribution differed sharply from the standard population, coverage probability in all but the Fay–Feuer method sometimes fell below 0.95. More efficient intervals than the Fay–Feuer interval were identified under specific circumstances. When the coefficient of variation of the age-adjustment weights was below 0.5, the Anderson–Rosenberg and Tiwari intervals appeared to be more efficient, whereas when it was above 0.5, the Fay–Kim interval appeared to be more efficient.

**Conclusions:**

As national and international agencies reassess prevailing data presentation standards to release age-adjusted estimates for smaller areas or population subgroups than previously presented, the Fay–Feuer interval can be used to develop interval-based statistical reliability standards with appropriate thresholds that are generally applicable. For data that meet certain statistical conditions, more efficient intervals could be considered.

## Background

The number of deaths reported for any given age group and time period can be assumed to follow a Poisson distribution, which leads to exact confidence intervals (CIs) for age-specific mortality rates [1, 2]. Further, because the sum of independent Poisson random variables is Poisson-distributed, the crude mortality rate also has an exact CI. However, no exact CI is known for age-adjusted mortality rates (i.e., directly standardized rates), because those are based on a *weighted* sum of Poisson random variables [3].

Various methods have been proposed to calculate approximate CIs for directly standardized rates, and recent simulation studies have continued to compare those methods based on metrics such as coverage probability and expected width; see [4–6] for three such simulation studies. To date, only the gamma-based method of Fay and Feuer [7] has been shown empirically to guarantee nominal coverage (e.g., 0.95 or higher probability that a 95% CI covers the true rate) in all simulation and real-world settings considered, though it often results in overly wide CIs. Tiwari et al. [8] developed a modification of the Fay–Feuer method to address the need for more efficient intervals (i.e., shorter intervals that retain nominal coverage) to accompany the estimates of age-adjusted rates that are published by the National Cancer Institute (NCI) at the US National Institutes of Health [9]. However, in some cases, CIs based on the Tiwari method can fail to retain nominal coverage [4]. Fay and Kim [10] proposed a mid-p modification to the Fay–Feuer CI, which does not guarantee nominal coverage but achieves it in many situations while remaining narrower than the Fay–Feuer or Tiwari CIs.

The Division of Vital Statistics at the US National Center for Health Statistics (NCHS), Centers for Disease Control and Prevention (CDC), had developed a gamma-based approximation to the Fay–Feuer method for the age-adjusted mortality rates that it published; see technical notes in Anderson and Rosenberg [11] as well as the methods section, below, for a description. Whereas the US Cancer Statistics (which include cancer registry data from NCI’s Surveillance, Epidemiology, and End Results Program as well as from CDC’s National Program of Cancer Registries) currently use the Fay–Feuer and Tiwari CIs [12], NCHS publications (e.g., National Vital Statistics Reports) and CDC WONDER use the Anderson–Rosenberg method when the number of events is less than 100; for 100 events or more, the normal CI is used [11, 13]. By design, the Anderson–Rosenberg method was simpler than the Fay–Feuer method to implement at NCHS as well as in the 57 state and local vital registration jurisdictions [14] because it could use pre-tabulated standard values for the upper and lower CI limits and allowed the user to more easily replicate calculations from published data.

With the wider availability of computing resources, the simplicity of the Anderson–Rosenberg method can no longer be the standalone rationale for its continued adoption in federal, state, or local agencies. Additionally, over the past 9 years, NCHS has been in the process of critically evaluating the hitherto prevailing statistical standards for the presentation of estimates in NCHS publications with an eye toward releasing statistically reliable estimates for sparse data (e.g., smaller geographical areas or population subgroups) that would have previously been suppressed due to sample size alone or other statistical considerations. Current statistical reliability standards for proportions at NCHS include sample-size based requirements (minimal sample or effective sample size) and interval-based criteria (thresholds for maximal length and relative width of “exact” confidence intervals) [15]. Similar criteria are under discussion for rates [16].

As of the writing of this manuscript, the prevailing NCHS standard for vital rates was sample size-based. Estimates would be suppressed or flagged as statistically unreliable if they were based on less than 20 events [17]. The interval-based thresholds discussed in [16] had not been adopted. For age-adjusted rates that are based on 20 or more events, and when the underlying at-risk population is large, the aforementioned gamma-based methods result in comparable CIs, all with at least nominal coverage, though, as will be seen below, the Anderson–Rosenberg CIs tend to be narrower (i.e., more efficient) than those from the original Fay–Feuer method and the Tiwari modification and can sometimes also be narrower than the Fay–Kim mid-p CIs. If the sample size threshold for presentation of rates was lowered from 20 to just 10 events, consistent with the minimum threshold required for disclosure protection of sub-national vitals data at NCHS [13] and elsewhere [18], but also with recent findings in [6] about the sufficient stability of estimates that are based on 10 events or more, then additional data presentation criteria could be required. If interval-based thresholds were to be used, then it would be necessary for the continued use of the Anderson–Rosenberg method to formally evaluate it in comparison with the other three gamma-based methods, specifically in terms of coverage probability and expected width, because, like the Tiwari and the Fay–Kim methods, it may also result in CIs that fail to maintain nominal coverage.

This paper conducts such a comparative evaluation, which, to our knowledge, had not previously been conducted. When data are sparse, our aim is to better understand the conditions that lead to the coverage probability of those a priori conservative CIs to fall below the desired level (for example, 0.95) or to CIs that are overly wide and less useful to assess precision. Our ultimate goal is to inform a CI-based statistical reliability threshold to use in conjunction with a sample size-based threshold of 10, say, as basis for the decision to suppress or present official estimates. Many other CI methods appear in the literature, and we do not aim to study them all here. We focus instead on the relative performance of the four aforementioned gamma-based methods because they are most relevant when a conservative approach to the assessment of statistical reliability of age-adjusted rates is desired.

## Methods

With *n* age groups, let *D*_*i*_ denote the number of deaths for group *i*. The *D*_*i*_ are assumed to be independent Poisson random variables, and the age-specific rates *R*_*i*_ are defined as the ratios *D*_*i*_/*P*_*i*_, with means ${\mathbb{E}}$(*R*_*i*_) = *λ*_*i*_ and variances ${\mathbb{V}}$(*R*_*i*_) = *λ*_*i*_/*p*_*i*_.

Let *π*_*i*_ denote the size of group *i* in the reference population; see "Technical Appendix". Let the *w*_*i*_ denote the relative proportions for group *i* in the reference population: *w*_*i*_ = *π*_*i*_/∑*π*_*j*_. The age-adjusted death rate *R′﻿﻿* is defined as

$$R^{\prime} = \sum w_{i} R_{i} = \sum \left( {w_{i} /P_{i} } \right)D_{i}$$

Given the parameters *λ*_*i*_ and denominators *P*_*i*_ = *p*_*i*_, the age-adjusted rate *R′* has mean ${\mathbb{E}}$(*R′*) = *λ′* = ∑*w*_*i*_* λ*_*i*_ and variance ${\mathbb{V}}$(*R′*) = ∑*w*_*i*_^2^ *λ*_*i*_/*p*_*i*_.

### Fay–Feuer interval

As explained in "Technical Appendix", Fay and Feuer [7] conjecture that tail probabilities for the age-adjusted rate *R′* can be approximated by those of a gamma-distributed random variable *Z* with ﻿${\mathbb{E}}$(*Z*) = *y* and ${\mathbb{V}}$﻿(*Z*) = *v*, i.e., with *α* = *y*^2^/*v* and *β* = *v*/*y*, where *y* = ∑(*w*_*i*_/*p*_*i*_) *x*_*i*_ and *v* = ∑(*w*_*i*_/*p*_*i*_)^2^ *x*_*i*_:

$${\text{Pr}} (R^{\prime} \ge y|\lambda^{\prime}) \approx \Pr (Z \le \lambda^{\prime}|y,v)$$

As a result, the lower limit *L*(*y*) of an equal-tailed 100(1 − *a*) percent CI for the parameter *λ′* can be resolved approximately from the lower tail probability of a gamma distribution with parameters *α* = *y*^2^/*v* and *β* = *v*/*y*, with the convention that *L*(0) = 0.

For the upper bound, the observed number of deaths *x*_*j*_ within group *j* is incremented by 1, resulting in the addition of the quantity *w*_*j*_/*p*_*j*_ to the age-adjusted rate *y* = ∑(*w*_*i*_/*p*_*i*_) *x*_*i*_. Because such a unit increment could be realized in any of the *n* groups,

$$\Pr [R^{\prime} > y|\lambda^{\prime} = U\left( y \right)\left] { \, \ge \, \Pr } \right[R^{\prime} \ge y + \kappa_{0} |\lambda^{\prime} = U\left( y \right)]$$

where *κ*_0_ = max{*w*_*j*_/*p*_*j*_}. Thus, an upper CI limit *U*(*y*) can be resolved from the upper tail probability of a gamma distribution with shape parameter *α* = *y′*^2^/*v′* and scale parameter *β* = *v′*/*y′* where *y′* = *y* + *κ*_0_ and *v′* = *v* + *κ*_0_^2^.

Fay and Feuer [7] conjecture that the approximate gamma CI thus constructed remains conservative. Although this conjecture remains unproven, findings from the many simulation studies to date continue to support it, e.g., [4–6].

### Tiwari modification

Tiwari et al. [8] developed a modification to the Fay–Feuer method described above by distributing an average increment 1/*n* uniformly across the *n* age groups instead of a unit increment in a single age group. Thus, with *κ*_1_ = *n *^−1^ ∑*w*_*i*_/*p*_*i*_ and *κ*_2_ = *n *^−1^ ∑(*w*_*i*_/*p*_*i*_)^2^, the gamma random variable *Z′* above now has mean *y′* = *y* + *κ*_1_ and variance *v′* = *v* + *κ*_2_. The Tiwari modification reduces the CI width relative to the Fay–Feuer method; see "Technical Appendix". However, the resulting CI sometimes fails to retain the nominal coverage level; see [4].

### Fay–Kim modification

Fay and Kim [10] more recently developed a mid-p version of the Fay–Feuer CI, as detailed in "Technical Appendix". Drawing *B* = *b* from a Bernoulli distribution with Pr(*B* = 1) = 1/2, the mid-p version uses the following gamma distribution:

$${\text{gamma}}_{{\text{mid-p}}} = b \times {\text{gamma}}\left( {y^{2} /v,v/y} \right) \quad\quad\quad\quad\quad\quad + \left( {1 - b} \right) \times {\text{gamma}}\left( {y^{{\prime}{2}} /v^{\prime},v^{\prime}/y^{\prime}} \right)$$

where﻿ *y′* = *y* + *κ*_0_ and *v′* = *v* + *κ*_0_^2^ are as in the Fay–Feuer construction. Thus, the lower and upper limits are defined as the (*a*/2)th and (1−*a*/2)th quantiles of gamma_mid-p_. R syntax is provided to find numerical solutions *L*(*y*) and *U*(*y*) [10].

### Anderson–Rosenberg approximation

Anderson and Rosenberg [11] had introduced an approximation to the Fay–Feuer upper CI limit that alleviated the need to calculate *κ*_0_ = max{*w*_*j*_/*p*_*j*_}; see "Technical Appendix". A “standardized” gamma random variable *G*_adj_ is defined as *Z*/(*v*/*y*), where the gamma-distributed *Z* has mean *y* and variance *v*. As a result, *G*_adj_ has mean and variance equal to *y*^*2*^/*v*. Define *x*_adj_ = *y*^2^/*v* and 1/*p*_adj_ = *v*/*y*. If *x*_adj_ was an integer, then there would exist a Poisson random variable *D*_adj_ with mean and variance equal to *λ′ p*_adj_ such that

$$\Pr \left( {D_{{{\text{adj}}}} \ge x_{{{\text{adj}}}} |\lambda^{\prime}} \right) = \Pr \left( {G_{{{\text{adj}}}} \le \lambda^{\prime}p_{{{\text{adj}}}} |x_{{{\text{adj}}}} } \right)$$

Because *y*^2^/*v* will generally not be integer, *x*_adj_ is defined as the nearest integer instead (although this is not strictly necessary), and the equality in this last equation is assumed to hold approximately. Either way, CI limits *L*(*y*) and *U*(*y*) for *λ′* are derived as the (*a*/2)-quantile of the gamma(*x*_adj_, 1/*p*_adj_) distribution and the (1 − *a*/2)-quantile of the gamma(*x*_adj_ + 1, 1/*p*_adj_), respectively.

### Comparisons among the four gamma-based CI methods

The Anderson–Rosenberg method can be seen not just as an approximation to, but as a modification of the Fay–Feuer CI that, like the Tiwari modification, reduces CI width. Further, a sufficient (but not necessary) condition exists that, when it holds, ensures the Anderson–Rosenberg CI is narrower than the Tiwari CI; see "Technical Appendix". Of course, as it is theoretically possible for both the Anderson–Rosenberg and the Tiwari CIs to be so narrow as to fail to retain nominal coverage, the empirical simulations, below, investigate situations where this may occur. In addition, these two CI methods are compared to the more recent Fay–Kim mid-p modification.

Several simulation scenarios were considered, each consisting of 500 simulations with 10,000 replicates. For each replication, the 95 percent CI limits were calculated according to the Fay–Feuer, Tiwari, Fay–Kim, and Anderson–Rosenberg methods. For each simulation, the coverage probability and expected CI width were tracked and plotted against the coefficient of variation (CV) of the weights *u*_*i*_ = *w*_*i*_/*p*_*i*_, as variability of the latter is known to contribute to under-coverage [7]. To account for simulation error, nominal 95% coverage was considered to have been achieved if the simulated coverage probability was ≥ 0.9449, which is the one-sided 99% confidence limit for a binomial with size 10,000 and success probability 0.95.

NCHS conventionally rounds the age-specific mortality rates, expressed as rates per 100,000 population, to one decimal point prior to calculating the age-adjusted rate for dissemination. In the simulations, unrounded values, including for *x*_adj_ and *p*_adj_, were retained for comparability with the other two gamma methods.

All simulations and data analyses were conducted in R version 4.1.2 [19].

#### Scenario 1

In the first set of simulations, counts were anchored to the median annual crude all-cause mortality rate in the USA from 2010 to 2019, estimated at 833.8 per 100,000 population, and the corresponding median annual probabilities of death in each age group, namely 0.009, 0.001, 0.002, 0.011, 0.018, 0.028, 0.066, 0.132, 0.181, 0.239, and 0.313 for < 1 year, 1–4, 5–14, 15–24, 25–34, 35–44, 45–54, 55–64, 65–74, 75–84, and 85 years and over, respectively. An overall population size of *P* = 2400 was selected to target a small overall mean number of events﻿ ${\mathbb{E}}$(*D*) = 20. The total number of deaths *D* was drawn from a Poisson distribution with mean﻿ ${\mathbb{E}}$(*D*). The age-specific event counts *D*_*i*_ were generated according to a multinomial distribution with ∑*D*_*i*_ = *D* and cell probabilities drawn from a Dirichlet distribution with concentration parameters equal to 833.8 times the above probabilities for each group. Finally, group sizes were generated according to a multinomial with ∑*P*_*i*_ = *P* and cell probabilities anchored at the median annual US values for the period 2010–2019, namely (0.012, 0.050, 0.129, 0.137, 0.137, 0.127, 0.135, 0.126, 0.084, 0.043, and 0.019) for the 11 age groups listed above.

Another simulation was conducted using the same scenario 1, but with a smaller target mean ﻿${\mathbb{E}}$(*D*) = 10. Here, because counts under 10 may be suppressed for disclosure protection (e.g., state- or county-level estimates in NCHS vital statistics releases), the statistical properties of CIs that accompany presented (non-suppressed) estimates will be impacted. Thus, a truncated Poisson distribution was used in the simulation to maintain the overall number of deaths ∑*D*_*i*_ = *D* ≥ 10, with the resulting true values of the crude, age-specific, and age-adjusted rates having been recalculated accordingly.

Because the year 2000 US standard population weights *w*_*i*_ were held constant and the age-specific population sizes *p*_*i*_ were generated in proportion to the overall US national age distribution, the CV for the weights *u*_*i*_ in scenario 1 remained in a relatively narrow range and was typically no larger than 0.30. To evaluate the performance of the four gamma CIs in situations where the weights *u*_*i*_ varied more widely, the settings in Fay and Feuer [7] were implemented, as described next.

#### Scenario 2

The second set of simulations mimicked the settings in [7] and [8], with the weights *u*_*i*_ = *w*_*i*_/*p*_*i*_ drawn at random from the uniform distribution on the unit interval. The total number of deaths *D* in the population was generated from a Poisson distribution with mean﻿ ${\mathbb{E}}$(*D*) = 20. The age-specific probabilities of death were drawn independently from the uniform distribution on the unit interval, and the counts *D*_*i*_ were drawn jointly from a multinomial distribution with ∑*D*_*i*_ = *D*. Again, to study the effect of a smaller overall mean number of events and assess the impact of disclosure protection on the statistical properties of CIs for estimates that are not suppressed, we also experimented with ${\mathbb{E}}$(*D*) = 10 using a truncated Poisson distribution to maintain ∑*D*_*i*_ = *D* ≥ 10.

#### Scenario 3

Finally, the gamma CI methods were evaluated in county-level mortality data from four causes of death, selected to capture varying age distributions: all causes; external causes of morbidity and mortality (ICD-10 codes: V01–Y89); congenital malformations, deformations, and chromosomal anomalies (Q00–Q99); and Alzheimer disease and other degenerative diseases of the nervous system, not elsewhere classified (G30–G31).

County-level data were queried using CDC WONDER as 20-year aggregate counts over the 1999–2019 period for 3147 US counties (boundary changes notwithstanding). Data were tabulated by age group (< 1 year, 1–4, 5–14, 15–24, 25–34, 35–44, 45–54, 55–64, 65–74, 75–84, and 85 years and over), race (American Indian or Alaska Native; Asian or Pacific Islander; Black or African American; and White), and Hispanic origin (Hispanic or Latino and not Hispanic or Latino).

Some counties had numerator case or population denominator counts under 10 for selected combinations of age and race and Hispanic origin, which were suppressed in CDC WONDER due to the NCHS confidentiality protection rules. Those missing cell case and/or population counts were imputed for this analysis, holding fixed the marginal counts by age and race and Hispanic origin, to obtain a complete, semi-synthetic dataset to use in simulations.

To investigate the impact of high CV on CI coverage for those sparse county-level data, each county's observed overall numerator count and age-adjusted death rate were taken as the “truth” and 10,000 replicates were generated according to a Poisson distribution for that county with the mean equal to the observed numerator count. The county’s overall population denominator was kept fixed. Age-specific numerator counts were assigned according to a multinomial distribution conditional on the crude total, with assignment probabilities for the various age groups taken proportional to the observed counts for that county. As in scenario 1, age-adjusted rates were calculated relative to the year 2000 US standard population.

The analyses for scenario 3 were restricted to data by race and Hispanic origin instead of sex or other demographic characteristics because disparities in health and mortality outcomes by race and Hispanic origin remain an important public health concern in the US [20], and because county-level estimates by race and Hispanic origin can be based on sparse data (less than 20 deaths) and suppressed or flagged as statistically unreliable in official publications when sample size is the only criterion used to define statistical reliability.

## Results

### Scenario 1

The top row in Fig. 1 shows the result of the first set of simulations, with the Fay–Feuer, Tiwari, and Anderson–Rosenberg CIs retaining nominal coverage over the limited range of variability of the weights *u*_*i*_ = *w*_*i*_/*p*_*i*_, whereas the coverage of the Fay–Kim CIs dipped below the 0.95 threshold in some cases, although those were within the simulation error bound of 0.9449. Additionally, the strength of the Anderson–Rosenberg approach is demonstrated in CIs that were consistently narrower (i.e., more efficient) than the Tiwari and Fay–Feuer CIs. The Fay–Kim method resulted in even narrower CIs for smaller CV values, albeit at the occasional cost of coverage probability falling below 0.9449.

**Fig.﻿ 1:**
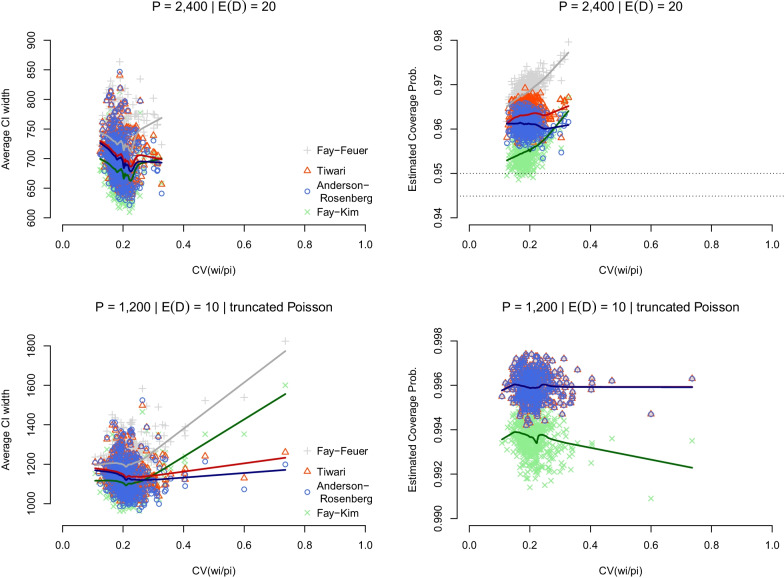
**Average width and coverage probability of selected gamma CIs for age-adjusted mortality rates: simulation scenario 1**. Average CI width and coverage probability of the Anderson–Rosenberg, Tiwari, Fay–Kim, and Fay–Feuer gamma CIs is based on 500 simulations with 10,000 replications each for age-adjusted mortality rates *R* = ∑(*w*_*i*_/*P*_*i*_) *D*_*i*_ and is presented as a function of the coefficient of variation (CV) of the weights *u*_*i*_ = *w*_*i*_/*p*_*i*_. Age-adjusted rates are anchored around an overall crude all-cause mortality rate of 833.8 per 100,000 population and the US national age distribution in 2010–2019. A multinomial distribution was used to generate the *D*_*i*_ conditional on the total *D* = ∑*D*_*i*_. Results in the top row are for an overall population size of *P* = 2400, corresponding to﻿ ${\mathbb{E}}$(*D*) = 20, whereas those in the bottom row are for *P* = 1200, corresponding to ${\mathbb{E}}$(*D*) = 10. For the latter, a truncated Poisson distribution was used in simulations to ensure the overall numerator count remained ≥ 10

The bottom row in Fig. 1 shows the results of a second set of simulations conducted using the same scenario 1, but with a smaller target mean ${\mathbb{E}}$(*D*) = 10 and a truncated Poisson distribution. The results here were similar to the ones in the top row of Fig. 1 and highlight the relative efficiency of the Anderson–Rosenberg CI, even in this sparser setting, compared with the Fay–Feuer and Tiwari methods for all values of CV(*u*_*i*_) shown, and with the Fay–Kim method for larger values of CV(*u*_*i*_).

### Scenario 2

The top row in Fig. 2 shows the result of the second set of simulations, which allow for an increased variability in the weights *u*_*i*_ = *w*_*i*_/*p*_*i*_, with both the Tiwari and Anderson–Rosenberg methods in close agreement and resulting in narrower intervals than the Fay–Feuer method while retaining 0.95 coverage, except for a handful of instances where the weights *u*_*i*_ = *w*_*i*_/*p*_*i*_ had CV close to 1.00. The Fay–Kim method performed relatively well when CV ≈ 1.00 compared to the Tiwari and Anderson–Rosenberg methods, increasing coverage probability (albeit with slightly wider CIs).

**Fig.﻿ 2:**
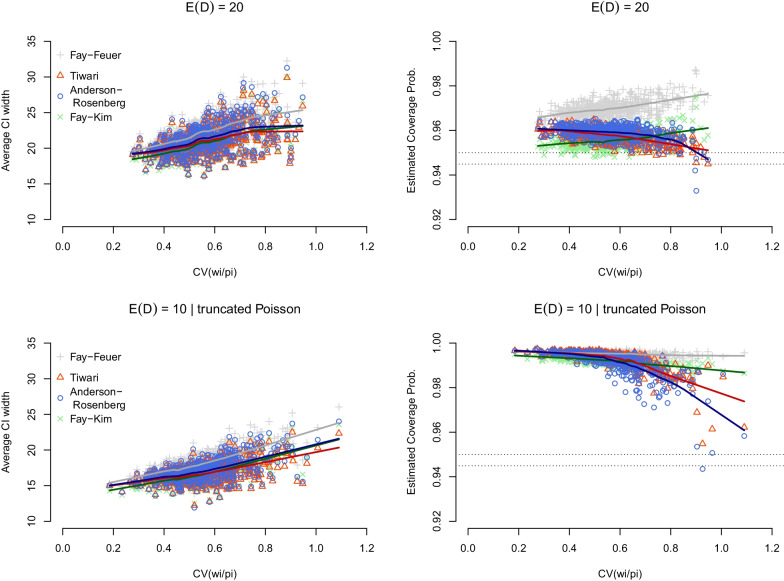
**Average width and coverage probability of selected gamma CIs for age-adjusted mortality rates: simulation scenario 2**. Average CI width and coverage probability of the Anderson–Rosenberg, Tiwari, Fay–Kim, and Fay–Feuer gamma CIs is based on 500 simulations with 10,000 replications each for age-adjusted mortality rates *R* = ∑(*w*_*i*_/*P*_*i*_) *D*_*i*_ and is presented as a function of the coefficient of variation (CV) of the weights *u*_*i*_ = *w*_*i*_/*p*_*i*_. The weights *u*_*i*_ and age-specific probabilities of death were drawn at random from the uniform distribution on the unit interval, and a multinomial distribution was used to generate the *D*_*i*_ conditional on the total *D* = ∑*D*_*i*_. In the top row, *D* was generated from a Poisson distribution with mean 20, whereas a mean of 10 was used in the bottom row. For the latter, a truncated Poisson distribution was used in simulations to ensure *D* remained ≥ 10

The bottom row in Fig. 2 shows the impact of a smaller target mean ${\mathbb{E}}$(*D*) = 10 and a truncated Poisson distribution, with the results that were again similar to the ones in the top row and showed adequate coverage for all three modifications to the original Fay–Feuer method, although coverage declined as weights variability increased.

### Scenario 3

Because high variability of the weights *w*_*i*_/*p*_*i*_ is a known contributor to under-coverage [7], as shown in Fig. 2, the distribution of these weights was examined using the county-level data, where the *p*_*i*_ are the age- and race- and Hispanic origin-specific population denominators for each county. Boxplots are shown in Fig. 3, with a CV as high as 3.0 for some counties and race and Hispanic origin groups.

**Fig. ﻿3:**
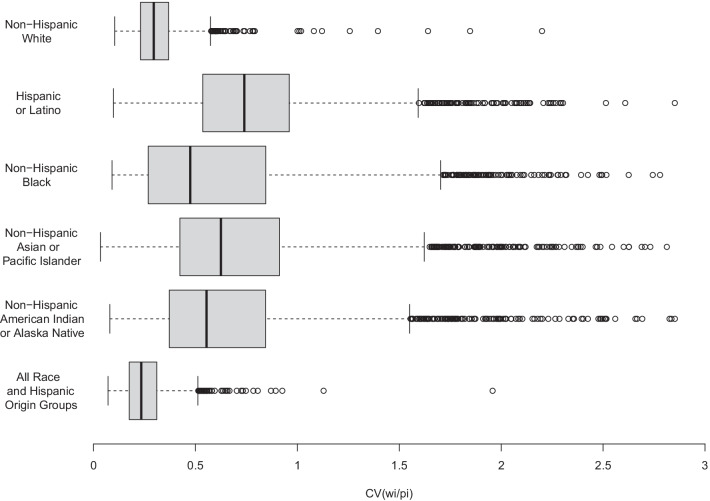
**Boxplots of county-level coefficients of variation for the weights ui, by race and Hispanic origin**. Boxplots of the coefficient of variation of the weights *u*_*i*_ = *w*_*i*_/*p*_*i*_ in the age-adjusted mortality rate *R* = ∑(*w*_*i*_/*P*_*i*_) *D*_*i*_ are presented by race and Hispanic origin, for 3147 US counties, using 1999–2019 aggregate data

Age-adjusted mortality rates for counties where the overall count *D* was less than 10 would be suppressed in accordance with NCHS confidentiality protection. Thus, comparisons among the four CI methods were most informative in counties with 10 or more deaths, as shown in Table 1. For those, when the CV of the *u*_*i*_ = *w*_*i*_/*p*_*i*_ was below 0.5, the Anderson–Rosenberg and Tiwari CIs almost always achieved nominal coverage, just like the Fay–Feuer CI, even for counties with 10–19 deaths. On the other hand, the Fay–Kim CI failed to achieve nominal coverage in cases where the other CIs did, notably for counties with 100 or more deaths. When the CV was larger than 0.5, there was a marked under-coverage for the Anderson–Rosenberg CIs, and, to a lesser extent, the Tiwari CI, in counties with 10–19 deaths but also in those with 20–99 deaths; in comparison, the Fay–Kim CI performed better in those cases, almost on par with the Fay–Feuer CI. Under-coverage of the Anderson–Rosenberg CI was more pronounced for rarer causes of death, e.g., ICD-10 codes Q00–Q99, in smaller and more clustered population subgroups than the non-Hispanic white population, such as the Hispanic or Latino or the non-Hispanic American Indian or Alaska Native populations, where nominal coverage was achieved for only about three in four counties with *D* = 10–99 and CV > 0.5.

**Table﻿ 1:** **Coverage of selected gamma CIs for county-level age-adjusted mortality rates, by race and Hispanic origin**. Coverage of the Anderson–Rosenberg, Tiwari, Fay–Kim, and Fay–Feuer gamma CIs is based on 10,000 replications for 3147 county-level age-adjusted mortality rates *R* = ∑(*w*_*i*_/*P*_*i*_) *D*_*i*_ by overall numerator size (*D* = ∑*D*_*i*_ < 10 vs. *D* = 10–19, *D* = 20–99, or *D* ≥ 100), coefficient of variation (CV) of the weights *u*_*i*_ = *w*_*i*_/*p*_*i*_ (CV > 0.5 vs. CV ≤ 0.5), and race and Hispanic origin for four causes of death, using 1999–2019 aggregate data. Causes of death include: all causes; external causes of morbidity and mortality (ICD-10 codes: V01–Y89); congenital malformations, deformations, and chromosomal anomalies (Q00–Q99); and Alzheimer disease and other degenerative diseases of the nervous system, not elsewhere classified (G30–G31)

		Counties with D < 10	Counties with D = 10–19		Counties with D = 20–99		Counties with D ≥ 100	
		(percent of total, n = 3147 counties)	CV ≤ 0.5 and nominal coverage achieved (% of total with CV ≤ 0.5)	CV > 0.5 and nominal coverage achieved (% of total with CV > 0.5)	CV ≤ 0.5 and nominal coverage achieved (% of total with CV ≤ 0.5)	CV > 0.5 and nominal coverage achieved (% of total with CV > 0.5)	CV ≤ 0.5 and nominal coverage achieved (% of total with CV ≤ 0.5)	CV > 0.5 and nominal coverage achieved (% of total with CV > 0.5)
*Anderson–Rosenberg CI*								
All-causes	American Indian or Alaska Native, Not Hispanic or Latino	1697 (53.9%)	243/247 (98.4%)	100/147 (68.0%)	380/380 (100%)	180/217 (82.9%)	282/282 (100%)	177/177 (100%)
	Asian or Pacific Islander, Not Hispanic or Latino	1673 (53.2%)	205/205 (100%)	171/236 (72.5%)	259/259 (100%)	288/302 (95.4%)	259/259 (100%)	213/213 (100%)
	Black or African American, Not Hispanic or Latino	823 (26.2%)	63/63 (100%)	132/207 (63.8%)	254/254 (100%)	239/260 (91.9%)	1298/1303 (99.6%)	237/237 (100%)
	Hispanic or Latino	599 (19.0%)	162/162 (100%)	345/443 (77.9%)	173/173 (100%)	716/741 (96.6%)	258/259 (99.6%)	770/770 (100%)
	White, Not Hispanic or Latino	1 (0.0%)	1/1 (100%)	0/2 (0.0%)	19/19 (100%)	5/8 (62.5%)	3020/3027 (99.8%)	89/89 (100%)
G30–G31	American Indian or Alaska Native, Not Hispanic or Latino	3026 (96.2%)	54/54 (100%)	25/25 (100%)	29/29 (100%)	11/11 (100%)	1/1 (100%)	1/1 (100%)
	Asian or Pacific Islander, Not Hispanic or Latino	2917 (92.7%)	52/52 (100%)	41/41 (100%)	67/67 (100%)	34/34 (100%)	34/34 (100%)	2/2 (100%)
	Black or African American, Not Hispanic or Latino	2084 (66.2%)	226/226 (100%)	37/37 (100%)	503/503 (100%)	49/49 (100%)	228/228 (100%)	20/20 (100%)
	Hispanic or Latino	2611 (83.0%)	52/52 (100%)	156/156 (100%)	72/72 (100%)	138/138 (100%)	52/52 (100%)	66/66 (100%)
	White, Not Hispanic or Latino	123 (3.9%)	108/108 (100%)	6/6 (100%)	795/795 (100%)	32/32 (100%)	2053/2053 (100%)	30/30 (100%)
Q00–Q99	American Indian or Alaska Native, Not Hispanic or Latino	3099 (98.5%)	16/16 (100%)	10/14 (71.4%)	10/10 (100%)	6/8 (75.0%)	0/0 (–)	0/0 (–)
	Asian or Pacific Islander, Not Hispanic or Latino	3018 (95.9%)	44/44 (100%)	25/28 (89.3%)	31/31 (100%)	15/15 (100%)	11/11 (100%)	0/0 (–)
	Black or African American, Not Hispanic or Latino	2597 (82.5%)	196/196 (100%)	28/37 (75.7%)	212/212 (100%)	30/35 (85.7%)	64/64 (100%)	6/6 (100%)
	Hispanic or Latino	2720 (86.4%)	40/41 (97.6%)	101/139 (72.7%)	40/40 (100%)	105/145 (72.4%)	23/23 (100%)	36/39 (92.3%)
	White, Not Hispanic or Latino	1047 (33.3%)	717/717 (100%)	12/12 (100%)	1060/1060 (100%)	11/11 (100%)	292/292 (100%)	8/8 (100%)
V01–Y89	American Indian or Alaska Native, Not Hispanic or Latino	2594 (82.4%)	115/115 (100%)	45/66 (68.2%)	156/156 (100%)	86/97 (88.7%)	58/58 (100%)	60/61 (98.4%)
	Asian or Pacific Islander, Not Hispanic or Latino	2593 (82.4%)	88/88 (100%)	86/124 (69.4%)	98/98 (100%)	102/115 (88.7%)	96/96 (100%)	33/33 (100%)
	Black or African American, Not Hispanic or Latino	1615 (51.3%)	162/163 (99.4%)	53/86 (61.6%)	511/511 (100%)	87/114 (76.3%)	580/581 (99.8%)	76/77 (98.7%)
	Hispanic or Latino	1657 (52.7%)	71/71 (100%)	251/375 (66.9%)	103/103 (100%)	383/533 (71.9%)	120/120 (100%)	280/288 (97.2%)
	White, Not Hispanic or Latino	22 (0.7%)	41/41 (100%)	5/11 (45.5%)	376/376 (100%)	31/35 (88.6%)	2610/2614 (99.8%)	48/48 (100%)
*Tiwari CI*								
All-causes	American Indian or Alaska Native, Not Hispanic or Latino	1697 (53.9%)	247/247 (100%)	111/147 (75.5%)	380/380 (100%)	180/217 (82.9%)	282/282 (100%)	176/177 (99.4%)
	Asian or Pacific Islander, Not Hispanic or Latino	1673 (53.2%)	205/205 (100%)	178/236 (75.4%)	259/259 (100%)	285/302 (94.4%)	259/259 (100%)	213/213 (100%)
	Black or African American, Not Hispanic or Latino	823 (26.2%)	63/63 (100%)	155/207 (74.9%)	254/254 (100%)	234/260 (90.0%)	1296/1303 (99.5%)	237/237 (100%)
	Hispanic or Latino	599 (19.0%)	162/162 (100%)	358/443 (80.8%)	173/173 (100%)	703/741 (94.9%)	258/259 (99.6%)	770/770 (100%)
	White, Not Hispanic or Latino	1 (0.0%)	1/1 (100%)	0/2 (0.0%)	19/19 (100%)	5/8 (62.5%)	3021/3027 (99.8%)	89/89 (100%)
G30–G31	American Indian or Alaska Native, Not Hispanic or Latino	3026 (96.2%)	54/54 (100%)	24/25 (96.0%)	29/29 (100%)	11/11 (100%)	1/1 (100%)	1/1 (100%)
	Asian or Pacific Islander, Not Hispanic or Latino	2917 (92.7%)	52/52 (100%)	40/41 (97.6%)	67/67 (100%)	34/34 (100%)	34/34 (100%)	2/2 (100%)
	Black or African American, Not Hispanic or Latino	2084 (66.2%)	226/226 (100%)	37/37 (100%)	503/503 (100%)	49/49 (100%)	228/228 (100%)	20/20 (100%)
	Hispanic or Latino	2611 (83.0%)	52/52 (100%)	153/156 (98.1%)	72/72 (100%)	138/138 (100%)	52/52 (100%)	66/66 (100%)
	White, Not Hispanic or Latino	123 (3.9%)	108/108 (100%)	6/6 (100%)	795/795 (100%)	32/32 (100%)	2053/2053 (100%)	30/30 (100%)
Q00–Q99	American Indian or Alaska Native, Not Hispanic or Latino	3099 (98.5%)	16/16 (100%)	12/14 (85.7%)	10/10 (100%)	7/8 (87.5%)	0/0 (–)	0/0 (–)
	Asian or Pacific Islander, Not Hispanic or Latino	3018 (95.9%)	44/44 (100%)	28/28 (100%)	31/31 (100%)	15/15 (100%)	11/11 (100%)	0/0 (–)
	Black or African American, Not Hispanic or Latino	2597 (82.5%)	196/196 (100%)	36/37 (97.3%)	212/212 (100%)	35/35 (100%)	64/64 (100%)	6/6 (100%)
	Hispanic or Latino	2720 (86.4%)	41/41 (100%)	134/139 (96.4%)	40/40 (100%)	140/145 (96.6%)	23/23 (100%)	39/39 (100%)
	White, Not Hispanic or Latino	1047 (33.3%)	717/717 (100%)	12/12 (100%)	1060/1060 (100%)	11/11 (100%)	292/292 (100%)	8/8 (100%)
V01–Y89	American Indian or Alaska Native, Not Hispanic or Latino	2594 (82.4%)	115/115 (100%)	61/66 (92.4%)	156/156 (100%)	94/97 (96.9%)	58/58 (100%)	60/61 (98.4%)
	Asian or Pacific Islander, Not Hispanic or Latino	2593 (82.4%)	88/88 (100%)	113/124 (91.1%)	98/98 (100%)	110/115 (95.7%)	96/96 (100%)	33/33 (100%)
	Black or African American, Not Hispanic or Latino	1615 (51.3%)	163/163 (100%)	79/86 (91.9%)	511/511 (100%)	106/114 (93.0%)	580/581 (99.8%)	77/77 (100%)
	Hispanic or Latino	1657 (52.7%)	71/71 (100%)	359/375 (95.7%)	103/103 (100%)	505/533 (94.7%)	120/120 (100%)	286/288 (99.3%)
	White, Not Hispanic or Latino	22 (0.7%)	41/41 (100%)	11/11 (100%)	376/376 (100%)	32/35 (91.4%)	2610/2614 (99.8%)	48/48 (100%)
*Fay–Kim CI*								
All-causes	American Indian or Alaska Native, Not Hispanic or Latino	1697 (53.9%)	247/247 (100%)	147/147 (100%)	380/380 (100%)	217/217 (100%)	281/282 (99.6%)	177/177 (100%)
	Asian or Pacific Islander, Not Hispanic or Latino	1673 (53.2%)	205/205 (100%)	236/236 (100%)	258/259 (99.6%)	302/302 (100%)	256/259 (98.8%)	213/213 (100%)
	Black or African American, Not Hispanic or Latino	823 (26.2%)	63/63 (100%)	207/207 (100%)	254/254 (100%)	260/260 (100%)	1289/1303 (98.9%)	237/237 (100%)
	Hispanic or Latino	599 (19.0%)	162/162 (100%)	443/443 (100%)	173/173 (100%)	741/741 (100%)	257/259 (99.2%)	768/770 (99.7%)
	White, Not Hispanic or Latino	1 (0.0%)	1/1 (100%)	2/2 (100%)	19/19 (100%)	8/8 (100%)	3004/3027 (99.2%)	88/89 (98.9%)
G30–G31	American Indian or Alaska Native, Not Hispanic or Latino	3026 (96.2%)	53/54 (98.1%)	25/25 (100%)	29/29 (100%)	11/11 (100%)	1/1 (100%)	1/1 (100%)
	Asian or Pacific Islander, Not Hispanic or Latino	2917 (92.7%)	52/52 (100%)	41/41 (100%)	67/67 (100%)	34/34 (100%)	33/34 (97.1%)	2/2 (100%)
	Black or African American, Not Hispanic or Latino	2084 (66.2%)	226/226 (100%)	37/37 (100%)	501/503 (99.6%)	49/49 (100%)	228/228 (100%)	20/20 (100%)
	Hispanic or Latino	2611 (83.0%)	46/52 (88.5%)	153/156 (98.1%)	71/72 (98.6%)	138/138 (100%)	52/52 (100%)	65/66 (98.5%)
	White, Not Hispanic or Latino	123 (3.9%)	108/108 (100%)	6/6 (100%)	794/795 (99.9%)	32/32 (100%)	2046/2053 (99.7%)	30/30 (100%)
Q00–Q99	American Indian or Alaska Native, Not Hispanic or Latino	3099 (98.5%)	16/16 (100%)	14/14 (100%)	10/10 (100%)	8/8 (100%)	0/0 (–)	0/0 (–)
	Asian or Pacific Islander, Not Hispanic or Latino	3018 (95.9%)	44/44 (100%)	28/28 (100%)	31/31 (100%)	15/15 (100%)	11/11 (100%)	0/0 (–)
	Black or African American, Not Hispanic or Latino	2597 (82.5%)	194/196 (99.0%)	37/37 (100%)	212/212 (100%)	35/35 (100%)	64/64 (100%)	6/6 (100%)
	Hispanic or Latino	2720 (86.4%)	41/41 (100%)	139/139 (100%)	40/40 (100%)	145/145 (100%)	23/23 (100%)	39/39 (100%)
	White, Not Hispanic or Latino	1047 (33.3%)	676/717 (94.3%)	12/12 (100%)	1059/1060 (99.9%)	11/11 (100%)	289/292 (99.0%)	8/8 (100%)
V01–Y89	American Indian or Alaska Native, Not Hispanic or Latino	2594 (82.4%)	115/115 (100%)	66/66 (100%)	156/156 (100%)	97/97 (100%)	57/58 (98.3%)	61/61 (100%)
	Asian or Pacific Islander, Not Hispanic or Latino	2593 (82.4%)	88/88 (100%)	124/124 (100%)	98/98 (100%)	115/115 (100%)	96/96 (100%)	33/33 (100%)
	Black or African American, Not Hispanic or Latino	1615 (51.3%)	163/163 (100%)	86/86 (100%)	511/511 (100%)	114/114 (100%)	580/581 (99.8%)	76/77 (98.7%)
	Hispanic or Latino	1657 (52.7%)	71/71 (100%)	375/375 (100%)	103/103 (100%)	533/533 (100%)	119/120 (99.2%)	287/288 (99.7%)
	White, Not Hispanic or Latino	22 (0.7%)	41/41 (100%)	11/11 (100%)	376/376 (100%)	35/35 (100%)	2592/2614 (99.2%)	47/48 (97.9%)
*Fay–Feuer CI*								
All-causes	American Indian or Alaska Native, Not Hispanic or Latino	1697 (53.9%)	247/247 (100%)	147/147 (100%)	380/380 (100%)	217/217 (100%)	282/282 (100%)	177/177 (100%)
	Asian or Pacific Islander, Not Hispanic or Latino	1673 (53.2%)	205/205 (100%)	236/236 (100%)	259/259 (100%)	302/302 (100%)	259/259 (100%)	213/213 (100%)
	Black or African American, Not Hispanic or Latino	823 (26.2%)	63/63 (100%)	207/207 (100%)	254/254 (100%)	260/260 (100%)	1299/1303 (99.7%)	237/237 (100%)
	Hispanic or Latino	599 (19.0%)	162/162 (100%)	443/443 (100%)	173/173 (100%)	741/741 (100%)	258/259 (99.6%)	770/770 (100%)
	White, Not Hispanic or Latino	1 (0.0%)	1/1 (100%)	2/2 (100%)	19/19 (100%)	8/8 (100%)	3022/3027 (99.8%)	89/89 (100%)
G30–G31	American Indian or Alaska Native, Not Hispanic or Latino	3026 (96.2%)	54/54 (100%)	25/25 (100%)	29/29 (100%)	11/11 (100%)	1/1 (100%)	1/1 (100%)
	Asian or Pacific Islander, Not Hispanic or Latino	2917 (92.7%)	52/52 (100%)	41/41 (100%)	67/67 (100%)	34/34 (100%)	34/34 (100%)	2/2 (100%)
	Black or African American, Not Hispanic or Latino	2084 (66.2%)	226/226 (100%)	37/37 (100%)	503/503 (100%)	49/49 (100%)	228/228 (100%)	20/20 (100%)
	Hispanic or Latino	2611 (83.0%)	52/52 (100%)	156/156 (100%)	72/72 (100%)	138/138 (100%)	52/52 (100%)	66/66 (100%)
	White, Not Hispanic or Latino	123 (3.9%)	108/108 (100%)	6/6 (100%)	795/795 (100%)	32/32 (100%)	2053/2053 (100%)	30/30 (100%)
Q00–Q99	American Indian or Alaska Native, Not Hispanic or Latino	3099 (98.5%)	16/16 (100%)	14/14 (100%)	10/10 (100%)	8/8 (100%)	0/0 (–)	0/0 (–)
	Asian or Pacific Islander, Not Hispanic or Latino	3018 (95.9%)	44/44 (100%)	28/28 (100%)	31/31 (100%)	15/15 (100%)	11/11 (100%)	0/0 (–)
	Black or African American, Not Hispanic or Latino	2597 (82.5%)	196/196 (100%)	37/37 (100%)	212/212 (100%)	35/35 (100%)	64/64 (100%)	6/6 (100%)
	Hispanic or Latino	2720 (86.4%)	41/41 (100%)	139/139 (100%)	40/40 (100%)	145/145 (100%)	23/23 (100%)	39/39 (100%)
	White, Not Hispanic or Latino	1047 (33.3%)	717/717 (100%)	12/12 (100%)	1060/1060 (100%)	11/11 (100%)	292/292 (100%)	8/8 (100%)
V01–Y89	American Indian or Alaska Native, Not Hispanic or Latino	2594 (82.4%)	115/115 (100%)	66/66 (100%)	156/156 (100%)	97/97 (100%)	58/58 (100%)	61/61 (100%)
	Asian or Pacific Islander, Not Hispanic or Latino	2593 (82.4%)	88/88 (100%)	124/124 (100%)	98/98 (100%)	115/115 (100%)	96/96 (100%)	33/33 (100%)
	Black or African American, Not Hispanic or Latino	1615 (51.3%)	163/163 (100%)	86/86 (100%)	511/511 (100%)	114/114 (100%)	580/581 (99.8%)	77/77 (100%)
	Hispanic or Latino	1657 (52.7%)	71/71 (100%)	375/375 (100%)	103/103 (100%)	533/533 (100%)	120/120 (100%)	288/288 (100%)
	White, Not Hispanic or Latino	22 (0.7%)	41/41 (100%)	11/11 (100%)	376/376 (100%)	35/35 (100%)	2611/2614 (99.9%)	48/48 (100%)

## Discussion

This paper conducted a comparative evaluation of four gamma-based methods for calculating CIs for age-adjusted mortality rates to inform their possible use in setting CI-based statistical reliability standards. In addition to being easier to implement because it can use pre-tabulated standard values for the upper and lower CI limits and allows the user to more easily replicate calculations from published data, the Anderson–Rosenberg CI appeared in simulations to be more efficient (i.e., shorter, while retaining nominal coverage) than either the Tiwari or Fay–Feuer CI in “large scale” estimates where the numerator count was large relative to the denominator population size and the age distribution followed the age distribution in the standard population. In contrast, even though the Fay–Kim method could result in even narrower CIs in those “large scale” scenarios, this was sometimes at the expense of the coverage probability falling below 0.95. However, for “small scale” estimates like county-level data by race and Hispanic origin for less common causes of death (scenario 3), and for which the age distribution differed sharply from the age distribution in the standard population, nominal CI coverage in both the Anderson–Rosenberg and Tiwari methods was compromised when the adjustment weights *u*_*i*_ = *w*_*i*_/*p*_*i*_ were highly variable, and the Fay–Kim method performed better in those situations, on par with the Fay–Feuer method. Nonetheless, in situations where the CV of the weights *u*_*i*_ = *w*_*i*_/*p*_*i*_ can be assessed in advance, when the CV is low, e.g., below 0.5, the user may still decide to use either the Anderson–Rosenberg or the Tiwari CIs instead of the Fay–Feuer CI if a shorter yet conservative interval is desired. If the user is willing to trade off sub-nominal coverage (e.g., coverage probability below 0.95 for 95% CIs) in some instances with low CV (e.g., below 0.5) for CIs that attain nominal coverage “on average” and are generally shorter, the Fay–Kim mid-p CI can be a good alternative.

## Conclusions

The Fay–Feuer CI can be used universally as the basis for formulating a CI-based threshold for statistical reliability of age-adjusted rates, because it maintains the nominal (e.g., 0.95 or higher) coverage probability in a large variety of studied situations. However, alternatives exist that are more efficient and perhaps more desirable under some specific circumstances. When the CV of the age-adjustment weights is below 0.5, the Anderson–Rosenberg and Tiwari CIs appear in simulations to be most efficient, whereas in cases where the CV is above 0.5, the Fay–Kim CI appears to be most efficient among the four gamma-based CI methods. In situations where the CV or the underlying distribution of the age-adjustment weights are unknown, while all four gamma-based methods studied in this paper appear to perform reasonably well, the Fay–Feuer method is recommended. For setting CI-based thresholds for statistical reliability, the properties of the interval can be considered, and thresholds for less efficient (wider) conservative intervals might be set higher than thresholds for more efficient (shorter) conservative intervals. However, it should be noted that such conservative CIs may have limited use in comparisons between two rates (e.g., by looking at whether there is overlap) because, as seen in simulations, they can be overly wide and will have low power to detect differences. Instead, differences in rates should be assessed using statistical significance testing or other suitable methods [21].

## Data Availability

The dataset analyzed during the current study is available from CDC WONDER, https://wonder.cdc.gov. The R syntax used is available from the authors on request.

## Technical appendix

The number of deaths *D* reported for a given area and time period is assumed to be Poisson-distributed, with mean ${\mathbb{E}}$(*D*) and variance ${\mathbb{V}}$(*D*) satisfying the equality ${\mathbb{E}}$(*D*) = *λP* = ${\mathbb{V}}$(*D*), where *P* denotes the population denominator [1]. The age-specific or crude death rate *R,* defined as the ratio *D*/*P,* is usually multiplied by 100,000 and reported as a rate per 100,000 population.

### Poisson-gamma relationship

For a positive integer *x* ≤ *P*, it can be shown [2] that there exists a gamma random variable *G* such that﻿ ${\mathbb{E}}$(*G*) = *x* =﻿ ${\mathbb{V}}$(*G*) and

A1 $$\Pr \left( {D \ge x|\lambda } \right) = \Pr \left( {G \le \lambda P|x} \right)$$

Recall that if *G* is gamma-distributed with shape parameter *α* > 0 and scale parameter *β* > 0, then its mean and variance are ${\mathbb{E}}$(*G*) = *αβ* and ${\mathbb{V}}$(*G*) = *αβ*^2^. Conversely, the parameters are given by *α* = ﻿${\mathbb{E}}$(*G*)^2^/${\mathbb{V}}$(*G*) and *β* = ${\mathbb{V}}$﻿(*G*)/${\mathbb{E}}$(*G*). Thus, with ﻿${\mathbb{E}}$(*G*) = *x* = ${\mathbb{V}}$(*G*) in Eq. A1, the corresponding gamma distribution has *α* = *x* and *β* = 1.

For the rate *R* = *D/P*, with *P* = *p*, *y* = *x*/*p*, and *v* = *x*/*p*^2^, Eq. A1 becomes

A2 $$\Pr (R \ge y|\lambda ) = \Pr (Z \le \lambda |y,v)$$

where *Z* = *G*/*P* is gamma-distributed with mean *y* and variance *v*.

### Gamma CI for age-specific and crude rates

When *D* = *x* is observed, the ratio *y* = *x*/*p* is an estimate of ${\mathbb{E}}$(*R*) = *λ*. An equal-tailed 100(1 − *a*) percent CI [*L*(*y*), *U*(*y*)] for the parameter *λ*, e.g., with *a* = 0.05, is obtained as a solution to the following two equations:

A3a $$\Pr \left[ {R \ge y|\lambda = L\left( y \right)} \right] = a/2$$

A3b $$\Pr \left[ {R \le y|\lambda = U\left( y \right)} \right] = a/2$$

Eqs. A3a and A3b follow from looking upon *L*(*y*) as the largest *λ* for which Pr(*R* ≥ *y*|*λ*) ≤ *a*/2 and *U*(*y*) as the smallest *λ* for which Pr(*R* ≤ *y*|*λ*) ≤ *a*/2; see [22] and theorem 9.2.3.a in [23].

From Eqs. A2 and A3a,

$$a/2 = \Pr [R \ge y|\lambda = L(y)] = \Pr [Z \le L\left( y \right)|y,v]$$

where *Z* is gamma-distributed with mean *y* and variance *v*, i.e., with parameters *α* = *y*^2^/*v* = *x* and *β* = *v*/*y* = 1/*p*. Thus, the lower CI limit *L*(*y*) is obtained as the (*a*/2)-quantile of the gamma(*x*, 1/*p*) distribution. For *y* = 0 = *x*, *L*(0) = 0 by convention.

Similarly, from Eqs. A2 and A3b,

$$1 - \left( {a/2} \right) = \Pr [R > y|\lambda = U(y)]  \quad\quad\quad\quad\, = \Pr [R \ge y + 1/p|\lambda = U(y)]  \quad\quad\quad\quad\, = \Pr [Z^{\prime} \le U(y)|y^{\prime},v^{\prime}]$$

where the second equality is due to *x* being a positive integer, so that *D*/*p* > *x*/*p* if and only if *D*/*p* ≥ (*x* + 1)/*p*, and *Z′* is a gamma random variable with mean *y′* = *y* + 1/*p* and variance *v′* = *v* + 1/*p*^2^. Because *y′*^2^/*v′* = *x* + 1 and *v′*/*y′* = 1/*p*, the upper CI limit *U*(*y*) is obtained as the (1 − *a*/2)-quantile of the gamma(*x* + 1, 1/*p*) distribution.

### Approximate gamma CIs for age-adjusted rates

With *n* age groups, let *D*_*i*_ denote the number of deaths for group *i*. The *D*_*i*_ are assumed to be independent Poisson random variables, and the age-specific rates *R*_*i*_ are defined as the ratios *D*_*i*_/*P*_*i*_, with means ${\mathbb{E}}$(*R*_*i*_) = *λ*_*i*_ and variances ${\mathbb{V}}$(*R*_*i*_) = *λ*_*i*_/*p*_*i*_.

Let *π*_*i*_ denote the size of group *i* in the reference population, e.g., the projected year 2000 US population [24]. Let *w*_*i*_ denote the relative proportions for group *i* in the reference population: *w*_*i*_ = *π*_*i*_/∑*π*_*j*_. The age-adjusted death rate *R′* is defined as

$$R^{\prime} = \sum w_{i} R_{i} = \sum \left( {w_{i} /P_{i} } \right)D_{i}$$

Given the parameters *λ*_*i*_ and denominators *P*_*i*_ = *p*_*i*_, the age-adjusted rate *R′* has mean ﻿${\mathbb{E}}$(*R′*) = *λ′* = ∑*w*_*i*_* λ*_*i*_ and variance ﻿${\mathbb{V}}$(*R′*) = ∑*w*_*i*_^2^ *λ*_*i*_/*p*_*i*_.

*Fay–Feuer interval.* Fay and Feuer [7] assume that Eq. A2 holds approximately for the age-adjusted rate *R′*, so that, for *y* = ∑(*w*_*i*_/*p*_*i*_) *x*_*i*_ and *v* = ∑(*w*_*i*_/*p*_*i*_)^2^ *x*_*i*_,

A4 $$\Pr (R^{\prime} \ge y|\lambda^{\prime}) \approx \Pr (Z \le \lambda^{\prime}|y,v)$$

where *Z* is gamma-distributed with ${\mathbb{E}}$(*Z*) = *y* and ${\mathbb{V}}$﻿(*Z*) = *v*, i.e., with *α* = *y*^2^/*v* and *β* = *v*/*y*. As for the crude rate *R*, an equal-tailed 100(1 − *a*) percent CI for *λ′* solves the equations:

A5a $$\Pr \left[ {R^{\prime} \ge y|\lambda^{\prime} = L\left( y \right)} \right] = a/2$$

A5b $$\Pr \left[ {R^{\prime} \le y|\lambda^{\prime} = U\left( y \right)} \right] = a/2$$

From Eqs. A4 and A5a, the lower limit *L*(*y*) can be resolved approximately from the lower tail probability of a gamma distribution with parameters *α* = *y*^2^/*v* and *β* = *v*/*y*, again with the convention that *L*(0) = 0.

For the upper bound, note that a unit increment in the observed number of deaths *x*_*j*_ within group *j* results in the addition of the quantity *w*_*j*_/*p*_*j*_ to the age-adjusted rate *y* = ∑(*w*_*i*_/*p*_*i*_) *x*_*i*_. Because such a unit increment could be realized in any of the *n* groups,

$$\Pr [R^{\prime} > y|\lambda^{\prime} = U(y)] \ge \Pr [R^{\prime} \ge y + \kappa_{0} |\lambda^{\prime} = U(y)]$$

where *κ*_0_ = max{*w*_*j*_/*p*_*j*_}. From Eq. A4, the right-hand side in this last inequality is approximately equal to Pr[*Z′* ≤ *U*(*y*)|*y′*, *v′*], where *Z′* is gamma-distributed with mean *y′* = *y* + *κ*_0_ and variance *v′* = *v* + *κ*_0_^2^. Thus, an upper CI limit *U*(*y*) can be resolved from the upper tail probability of a gamma distribution with shape parameter *α* = *y′*^2^/*v′* and scale parameter *β* = *v′*/*y′*. Fay and Feuer [7] make the conjecture that the approximate gamma CI thus constructed remains conservative. Although this conjecture remains unproven, findings from the many simulation studies to date continue to support it, e.g., [4–6].

*Tiwari modification.* Tiwari et al. [8] developed a modification to the Fay–Feuer method described above by distributing an average increment 1/*n* uniformly across all age groups instead of a unit increment in a single age group:

$$y^{\prime} = \sum \left( {w_{i} /p_{i} } \right)\left( {x_{i} + \frac{1}{n}} \right) = y + \frac{1}{n}\sum w_{i} /p_{i}$$

Thus, with *κ*_1_ = *n*^−1^ *∑w*_*i*_/*p*_*i*_ and *κ*_2_ = *n*^−1^ ∑(*w*_*i*_/*p*_*i*_)^2^, the gamma random variable *Z′* above now has mean *y′* = *y* + *κ*_1_ and variance *v′* = *v* + *κ*_2_. The Tiwari modification reduces the CI width relative to the Fay–Feuer method, because

$$\Pr [R^{\prime} \ge y + \kappa_{1} |\lambda^{\prime} = U(y)] \ge \Pr [R^{\prime} \ge y + \kappa_{0} |\lambda^{\prime} = U(y)]$$

However, the resulting CI sometimes fails to retain the nominal coverage level, e.g., [4].

*Fay–Kim modification.* Fay and Kim [10] more recently developed a mid-p version of the Fay–Feuer CI. A modification of exact CIs from discrete data, mid-p CIs trade-off guaranteed nominal coverage in all of the parameter space (which tends to result in overly wide CIs) for proximity to nominal coverage (and narrower CIs) for most parameter values.

For the mid-p interval, a solution to the following equations is sought:

A7a $$\Pr \left[ {R^{\prime} > y|\lambda^{\prime} = L\left( y \right)} \right] \quad\quad\quad\quad + \left( {1/2} \right) \times \Pr \left[ {R^{\prime} = y|\lambda^{\prime} = L\left( y \right)} \right] = a/2$$

A7b $$\Pr \left[ {R^{\prime} < y|\lambda^{\prime} = U\left( y \right)} \right] \quad\quad\quad\quad + \left( {1/2} \right) \times \Pr \left[ {R^{\prime} = y|\lambda^{\prime} = U\left( y \right)} \right] = a/2$$

Drawing *B* = *b* from a Bernoulli distribution with Pr(*B* = 1) = 1/2, Fay and Kim [10] define the mid-p version of the Fay–Feuer CI using the following gamma distribution:

$${\text{gamma}}_{{\text{mid-p}}} = b \times {\text{gamma}}\left( {y^{2} /v,v/y} \right) \quad\quad\quad\quad\quad\quad + \left( {1 - b} \right) \times {\text{gamma}}\left( {y^{{\prime}{2}} /v^{\prime},v^{\prime}/y^{\prime}} \right)$$

where *y′* = *y* + *κ*_0_ and *v′* = *v* + *κ*_0_^2^ are as in the Fay–Feuer construction, above. Thus, the lower and upper limits are defined as the (*a*/2)th and (1−*a*/2)th quantiles of gamma _mid-p_. The special case *y* = 0 is addressed using *L*(0) = 0 and *U*(0) defined as the (1−*a*)th quantile of the gamma(*y*^2^/*v, v*/*y*) distribution. R syntax is provided to solve for *L*(*y*) and *U*(*y*) numerically [10].

*Anderson–Rosenberg approximation.* Anderson and Rosenberg [11] had introduced an approximation to the Fay–Feuer upper CI limit that alleviated the need to calculate *κ*_0_ = max{*w*_*j*_/*p*_*j*_}. Instead, the Poisson-gamma relationship in Eq. A1 is assumed to hold for an appropriately defined Poisson random variable *D*_adj_ corresponding to a crude rate that would have been equal to the age-adjusted rate *R′*, i.e., such that *R′* = *D*_adj_/*P*_adj_. Therefore, a “standardized” gamma random variable *G*_adj_ is defined as *Z*/(*v*/*y*), where the gamma-distributed *Z* has mean *y* and variance *v*. As a result, *G*_adj_ has mean and variance equal to *y*^*2*^/*v*. Define *x*_adj_ = *y*^2^/*v* and 1/*p*_adj_ = *v*/*y*. If *x*_adj_ was an integer, then there would exist a Poisson random variable *D*_adj_ with mean and variance equal to *λ′ p*_adj_ such that

A6 $$\Pr \left( {D_{{{\text{adj}}}} \ge x_{{{\text{adj}}}} |\lambda^{\prime}} \right) = \Pr \left( {G_{{{\text{adj}}}} \le \lambda^{\prime}p_{{{\text{adj}}}} |x_{{{\text{adj}}}} } \right)$$

Because *y*^2^/*v* will generally not be integer, *x*_adj_ is defined as the nearest integer instead (although this is not strictly necessary), and the equality in Eq. A6 is assumed to hold approximately. Either way, one proceeds as for the crude rate to derive CI limits *L*(*y*) and *U*(*y*) for *λ′* as the (*a*/2)-quantile of the gamma(*x*_adj_, 1/*p*_adj_) distribution and the (1 − *a*/2)-quantile of the gamma(*x*_adj_ + 1, 1/*p*_adj_), respectively.

*Exact intervals.* When there is a constant scalar *c* > 0 such that *p*_*i*_ = *cπ*_*i*_ for all *i*, the age-adjusted rate equals the overall crude rate, and the above CIs reduce to the exact gamma CI for *λ* = *p*^*−*1^ ∑*λ*_*i*_* p*_*i*_ where *p* = ∑*p*_*i*_ and the total number of deaths *D* = ∑*D*_*i*_ follows a Poisson distribution with mean *λp*. In particular, when *y* = 0, *v* = 0 and *x*_adj_ is undefined. However, because the age-adjusted rate equals the crude rate in this case, the limits of all three approximate gamma CIs for the age-adjusted rate are defined to be those of the exact gamma CI for the crude rate, with *p* = ∑*p*_*i*_ and *x* = ∑*x*_*i*_ = 0. Thus, in this extreme case, *L*(0) = 0 and *U*(0) is the (1 − *a*/2)-quantile of the gamma(1, 1/*p*) distribution.

*Anderson–Rosenberg CI as a modification of the Fay–Feuer CI.* The Anderson–Rosenberg construction can be seen to follow that of the Fay–Feuer CI, with a gamma-distributed *Z′′* that has mean *y′′* = *y* + *κ* and variance *v′′* = *v* + *κ*^2^, where *κ* = *κ*_3_ = 1/*p*_adj_ instead of* κ* = *κ*_0_ = max{*w*_*j*_/*p*_*j*_}. Indeed, with 1/*p*_adj_ = *v*/*y* and *x*_adj_ = *y*^2^/*v*,

$$\frac{{v^{\prime\prime} }}{{y^{\prime\prime} }} = \frac{{v(y^{2} + v)/y^{2} }}{{(y^{2} + v)/y}} = \frac{v}{y} = \frac{1}{{p_{{{\text{adj}}}} }}$$

$${\text{and}}\quad \frac{{y^{{\prime\prime}{2}} }}{{v^{\prime\prime}}} = \frac{{(y^{2} + v)^{2} /y^{2} }}{{v(y^{2} + v)/y^{2} }} = \frac{{y^{2} + v}}{v} = x_{{{\text{adj}}}} + 1$$

Furthermore, 1/*p*_adj_ can be expressed as follows:

$$\begin{aligned}\frac{1}{{p_{{{\text{adj}}}} }} &= \frac{v}{y} = \sum \left( {w_{i} /p_{i} } \right)\xi_{i} \quad{\text{with}}\\\xi_{i} &= \frac{{(w_{i} /p_{i} )x_{i} }}{{\sum (w_{j} /p_{j} )x_{j} }}\;{\text{and}}\;\sum \xi_{i} = 1.\end{aligned}$$

As a result,

$$y^{\prime\prime} = y + \frac{1}{{p_{{{\text{adj}}}} }} = \sum (w_{i} /p_{i} )(x_{i} + \xi_{i} )$$

and ﻿the Anderson–Rosenberg method is seen to result in incrementing the age-specific death counts from *x*_*i*_ to *x*_*i*_ + *ξ*_*i*_, whereas in the Fay–Feuer method only the count *x*_*i**_ for the age group *i** for which *w*_*i**_/*p*_*i**_ = max{*w*_*j*_/*p*_*j*_} is incremented—and in the Tiwari modification, the age-specific counts are incremented from *x*_*i*_ to *x*_*i*_ + *ζ*_*i*_, where *ζ*_*i*_ = 1/*n*. Additionally,

$$\kappa_{3} = \frac{1}{{p_{{{\text{adj}}}} }} = \sum (w_{i} /p_{i} )\xi_{i} \le \max \{ w_{i} /p_{i} \} = \kappa_{0}$$

since ﻿∑*ξ*_*i*_ = 1. Thus, like the Tiwari modification, the Anderson–Rosenberg construction reduces the CI width relative to the Fay–Feuer method:

$$\Pr [R^{\prime} \ge y + \kappa_{3} |\lambda^{\prime} = U(y)] \ge \Pr [R^{\prime} \ge y + \kappa_{0} |\lambda^{\prime} = U(y)]$$

Two questions emerge from the above derivations:

1. Under what circumstances does the Anderson–Rosenberg method result in a shorter CI than the Fay–Feuer method that retains nominal coverage?
2. Since both the Anderson–Rosenberg and Tiwari methods result in narrower CIs than the Fay–Feuer method, when is one preferable to the other?

To partially answer question 2, note that the Anderson–Rosenberg CI would be narrower than the Tiwari CI if (but not only if) *κ*_3_ ≤ *κ*_1_, as that ensures

$$\Pr [R^{\prime} \ge y + \kappa_{3} |\lambda^{\prime} = U(y)] \ge \Pr [R^{\prime} \ge y + \kappa_{1} |\lambda^{\prime} = U(y)]$$

By definition, the condition *κ*_3_ ≤ *κ*_1_ is realized when

$$\sum (w_{i} /p_{i} )\xi_{i} \le \frac{1}{n}\sum (w_{i} /p_{i} )$$

﻿which is equivalent to

$$\frac{1}{n}\sum (w_{i} /p_{i} )^{2} x_{i} \le \left\{ {\frac{1}{n}\sum (w_{i} /p_{i} )x_{i} } \right\} \left\{ {\frac{1}{n}\sum (w_{i} /p_{i} )} \right\}$$

This last condition indicates that the slope of the line from the simple regression of the weight-adjusted age-specific death rates *w*_*i*_ (*x*_*i*_/*p*_*i*_) = (*w*_*i*_/*p*_*i*_) *x*_*i*_ onto the weights *w*_*i*_/*p*_*i*_ is negative or zero. This could be verified upfront for any set of age-adjustment weights (*w*_1_, …, *w*_n_) and population distribution (*p*_1_, …, *p*_n_), and it would be sufficient to ensure that the Anderson–Rosenberg CI will be narrower than the Tiwari CI. Of course, this leaves the issue of efficiency unresolved, as it would theoretically be possible for either the Anderson–Rosenberg or the Tiwari CIs to be so narrow as to fail to retain nominal coverage. The empirical simulations investigate situations where this may occur. In addition, these two CI methods are compared to the more recent Fay–Kim mid-p modification.

## Abbreviations

CI: Confidence interval
NCHS: National Center for Health Statistics
NCI: National Cancer Institute
CDC: Centers for Disease Control and Prevention
ICD-10: International Classification of Diseases, Tenth Revision
